# The Evolution of African Swine Fever in China: A Global Threat?

**DOI:** 10.3389/fvets.2022.828498

**Published:** 2022-03-29

**Authors:** Satoshi Ito, Jaime Bosch, Marta Martínez-Avilés, José Manuel Sánchez-Vizcaíno

**Affiliations:** ^1^VISAVET Health Surveillance Center, Complutense University of Madrid, Madrid, Spain; ^2^Department of Animal Health, Faculty of Veterinary, Complutense University of Madrid, Madrid, Spain; ^3^Infectious Diseases and Global Health Department, Centro de Investigación en Sanidad Animal (CISA), Instituto Nacional de Investigación y Tecnología Agraria y Alimentaria-Consejo Superior de Investigaciones Científicas (INIA-CSIC), Madrid, Spain

**Keywords:** spatio-temporal epidemic modeling, infectious diseases, African swine fever, risk assessment, Asia, China, veterinary epidemiology, lower virulent ASFV

## Abstract

African swine fever (ASF) is one of the most critical diseases in the pig industry. In Asia, 15 countries have already reported an outbreak as of November 22, 2021. In 2021, China reported the genotype II lower virulent ASF virus (ASFV) and the emergence of genotype I ASFV. ASF is generally known as a contagious and lethal disease, but if chronic infection spreads, then disease control would be more difficult. In the current study, we highlighted the possibility of lower virulent virus distribution throughout China and the subsequent general risk of the virus being released from the country. The kernel density estimation showed that the two highest kernel density areas of ASF notification were located in Northeast and Midwest China. Four of the five provinces where lower virulent ASFV was isolated overlapped with areas of relatively high ASF notification density. In terms of the risk of ASFV spreading from China, eight of the 10 largest airports and three of the 10 largest seaports are located in areas of relatively high ASF notification density. There were flight flow from China to 67 countries and ship flow to 81 countries. Asia had the highest flight flow, followed by Europe, North America, Africa, and Oceania. The highest number of ship flows was also concentrated in Asia, but about 10% of ships head to Africa and South America. Chinese overseas residents were distributed in each continent in proportion to these results. Here, we highlight the potential risk of ASFV spread from China to the world.

## Introduction

African swine fever (ASF) is one of the most feared diseases in the pig industry in recent years. This devastating transboundary disease is caused by the ASF virus (ASFV), and pig species are the only susceptible animal population. On the basis of the p72 genotyping classification, 24 genotypes have been reported worldwide to date (1, 2). Of these viruses, the ones that are currently widely spread throughout Europe and Asia are genotype II viruses, which are generally regarded as highly virulent (3). The clinical signs exhibited by infected individuals vary and are classified into four main stages based on clinical presentations and pathological lesions: Peracute, Acute, Subacute, and Chronic stage (4). Subacute and chronic forms of ASF have a low mortality rate in infected individuals. In particular, chronically infected individuals show unclear clinical symptoms, and some individuals have been reported to spread the virus for a long time (4). Susceptible individuals can become infected with the virus *via* direct or indirect contact with infected animals or contaminated materials (5, 6).

ASFV is well-known as a virus with a significantly high environmental resistance. Various studies have been conducted on the environmental resistance of viruses. The virus is shed in large quantities in the blood where the virus can survive for 15 weeks at room temperature, months at 4°C, and indefinitely when frozen (7). In the case of raw meat, it can survive for more than 3 months in meat and offal [FAO, (5, 7)]. Feces and urine are also infectious. The half-life of the virus in urine is 15 days depending on the environmental temperature (8). In feces, its half-life is reported to be 5–8 days, but viral DNA can be detected for up to 2–4 years (8). Therefore, any contaminated material, including persons, materials, or infected meat products, could represent a source of infection to ASF-free countries. Even in countries where outbreaks have already been reported, a higher level of environmental contamination could pose a higher risk of further outbreaks.

In Asia, after the first ASF outbreak was reported in Liaoning Province, China, in August 2018, 15 countries (China, Mongolia, Vietnam, Cambodia, North Korea, Laos, Myanmar, Philippines, South Korea, East Timor, Indonesia, Papua New Guinea, India, Malaysia, and Bhutan) confirmed ASF outbreaks as of November 22, 2021 (9). Although many ASF cases have been reported in wild boar in South Korea and Malaysia, ASF outbreaks in the remaining Asian countries have been mainly associated with domestic pigs at this moment (10). This is assumed to be related to the traditionally high number of backyard farms, inadequate biosecurity levels, and the non-transparent distribution network of livestock and their products (11).

Although ASFV genotype II is currently prevalent in Asia and is considered to be highly virulent, the isolation of the lower virulent ASFV genotype I and II was recently reported in China (12, 13). By deleting genes, ASFV can be artificially attenuated (14). The *EP402R* gene encodes the CD2v protein, which causes binding of red blood cells to infected cells and virus particles (3, 15). The deletion of this gene resulted in virus attenuation and induction of protection, so it is frequently targeted for ASF vaccine development (15–17). Recently, there has been a problem in China with the spread of illegal vaccines created by copying vaccines under development (14, 18). These illegal vaccines might cause chronic infection in vaccinated individuals (14). Apparently supporting this fact, Sun et al. surveyed seven Chinese provinces (Heilongjiang, Jilin, Liaoning, Shanxi, Inner Mongolia Autonomous Region, Hebei, and Hubei) with the collection of 3,660 field samples in 2020 (13), and they detected 11 different isolates of CD2v (–) ASFV of genotype II, which show lower virulence, in three provinces (seven from Heilongjiang, three from Hubei, and one from Hebei). Moreover, in June 2021, Sun et al. reported the emergence of ASFV genotype I, for the first time in Asia, from two farms in Henan and Shandong province, China (12). The results of animal experiments have shown that these isolated viruses [ASFV genotype I and CD2v (–) ASFV genotype II] can cause chronic infection and are highly transmissible (12, 13). These lower virulent viruses are characterized by unclear clinical symptoms and a long incubation period, which makes early detection of infected animals more difficult (12, 13). If these lower virulent viruses are quietly spreading throughout China and are released from the country, then this will most likely further complicate any efforts to control ASF in affected countries.

In this study, we first highlight the spatial distribution of ASF notifications across China. Second, the spatial risk of these lower virulent ASFVs [ASFV genotype I and CD2v (–) ASFV genotype II], spreading throughout the country, was assessed. To do so, we overlaid the ASF notification density map with the areas where these lower virulent viruses were found, then we indicate, in an indirect manner, the general risk of ASFV spread from China.

## Materials and Methods

### Data and Sources

The whole of China was set as the study area. Epidemiological information regarding dates, coordinates, and event source (fomites, swill feeding, illegal movement of animals, etc.) including both domestic pig and wild boar for the periods from August 1, 2018, to September 4, 2021, were obtained from the World Animal Health Information System (OIE-WAHIS) database (9).

### Temporal Trend and Spatial Distribution of ASF in China

On the basis of the information obtained from OIE-WAHIS (9), 3-month outbreak trends for ASF in China, including epidemic information (reported number and event sources), were described using Microsoft Excel Software. Furthermore, each outbreak was categorized by year, by event source, and its spatial distribution was depicted using ArcGIS 10.8.1 software (ESRI Inc., Redlands, CA, USA).

### Risk of the Lower Virulent ASFV Spreading Nationwide

To assess the spatial risk of ASF outbreaks spread across the country, the kernel density analysis (19), a non-parametric estimation tool for assuming the continuous density distribution from a series of events, was applied using the data obtained from OIE-WAHIS (9, 20). Here, we assumed that if the lower virulent ASFV is located in an area of high kernel density, the risk of the virus being spread is considered as high. For more accurate kernel density maps, it is important to obtain the correct location of the case point and to set the search radius (bandwidth) appropriately. To explore the ideal bandwidth, we applied a multi-distance spatial cluster analysis tool in ArcGIS software version 10.8.1 according to the manufacturer's guidelines and previous studies (21, 22). A common transformation of Ripley's K function was used in the analysis. For analysis of the spatial pattern of ASF outbreaks, observed K values were compared with the Expected K values of a completely random spatial distribution of ASF outbreaks with 999 simulations, which is equal to a confidence level of 99.9%. The Diff K values contain the Observed K values minus the Expected K values. The Expected K values giving the highest Diff K values can be interpreted as the maximum distance of the relationships between ASF outbreaks in China; thus, it was set as the optimal bandwidth. The kernel densities obtained were then classified into five risk levels based on Jenks natural brakes classification (23) and defined as very high, high, medium, low, and very low as indirect risk indicators.

In general, the size of the pig production in the area is considered as one of the important factors that affect the occurrence of ASF (24). To visually understand the relationship between the kernel density of ASF notification and the scale of the swine industry, we firstly identified provinces with high pig stocks on average based on information from the Global Economic Data, Indicators, Charts & Forecasts (CEIC) database for 2017–2019 (25) and then overlaid these locations on the map. Furthermore, to investigate the possibility that the lower virulent ASFV is spreading throughout China, the results of the kernel density analysis and the location of the provinces where these viruses have been isolated (12, 13) were depicted together on the map using ArcGIS 10.8.1 software. In this study, lower virulent ASFV was defined as ASFV genotype I and CD2v (–) ASFV genotype II isolated in China.

### The Risk of ASFV Spread From China

In this section, we assessed the general risk of ASFV spread from China. There are various pathways by which the virus can be released from the country. These include the movement of live pigs, pork products, and human movement such as travel or humanitarian aid (26). China has not exported live pigs to foreign countries since the first confirmation of ASF in China, and the number of destinations for pork products is very limited (27). Therefore, the most probable route of virus release is assumed to be *via* international travel by ship or airplane.

Because there was insufficient information available to conduct a quantitative risk assessment in this study, the risk was estimated indirectly by considering the flight and ship flow from China and the number of Chinese overseas residents per country.

First, to understand the geographical relationship between the ASF confirmed area and international ports, the locations of China's 10 largest airports and seaports (28, 29) were overlaid with the map of kernel density analysis using ArcGIS 10.8.1 software. Second, information on traffic volumes at ports trading with China was obtained from the World Bank database (30) and converted to national scale information using Microsoft Excel Software.

For flight flow, airports with flights coming from China were attributed with total seats from the year 2019. Regarding ship flow, we calculated the loading capacity between China and the destination countries. All seaports with reported international trade in the first quarter (Q1) of 2020 were attributed with the sum of quarterly deployed capacity twenty-foot equivalent unit (TEU) (31, 32). The obtained results were depicted on the world maps using the XY To Line tool on ArcGIS 10.8.1 software (33).

According to previous studies, Chinese tourists tend to prefer Chinese food at least once a day even when they are in a travel destination, and, even after several generations of migration abroad, they still have the habit of eating Chinese food on a daily basis (34–36). In this study, the Chinese overseas residents were defined as people of Chinese birth or ethnicity who reside outside China. We assumed that Chinese overseas residents may bring in goods from China, which could be one of the important risky behaviors for other countries in terms of ASFV introduction. At present, there is no official information on the number of Chinese overseas residents. Therefore, the available relevant information was collected on the basis of various data sources (37–41). The obtained data were depicted by overlaying a map showing the flight or ship flows from China using the Add Join tool on ArcGIS 10.8.1 software (42).

## Results

### Temporal Trend and Spatial Distribution of ASF in China

**Figure 1:**
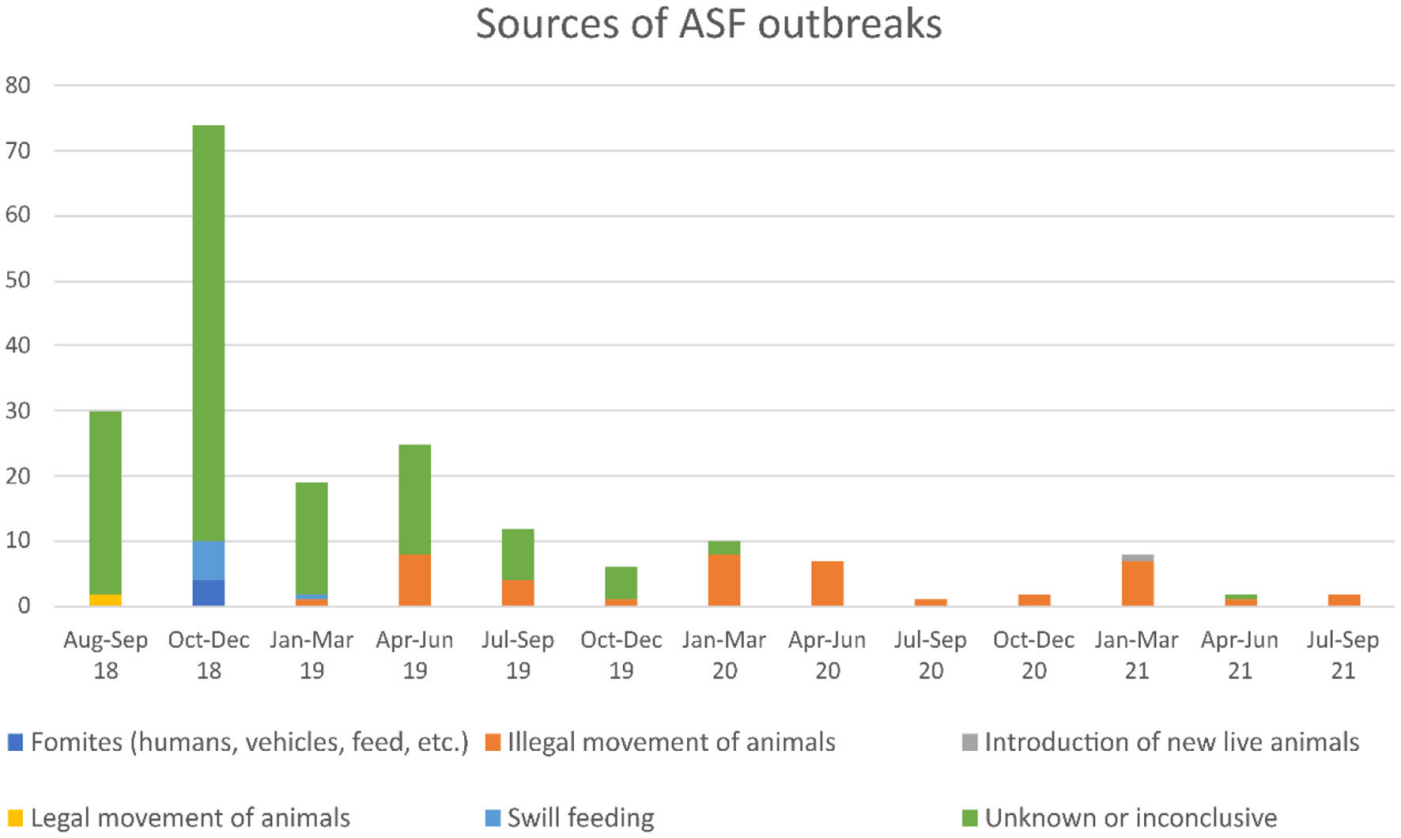
Temporal trend of African swine fever (ASF) in China. The number of ASF outbreaks every 3 months, including the details of the event sources, is shown in a bar graph. These information sources are based on official reports to the OIE.

The 3-month temporal trend of ASF outbreaks is shown in Figure 1. After a peak in the number of outbreaks in the last quarter of 2018, there was an overall decreasing trend in the number of notifications. In the third quarter of 2020, there were only three outbreaks, but the ongoing outbreaks continued, and since then, the number of outbreaks increased again. Focusing on the characteristics of each outbreak, the sources of outbreaks reported to the OIE show differences in 2018–2019 and in 2020–2021. About 83.6% of the event sources of ASF reported from 2018 to 2019 were “unknown or inconclusive”. However, after 2020, about 87.5% of the event sources were “illegal transport of animals”.

**Figure 2:**
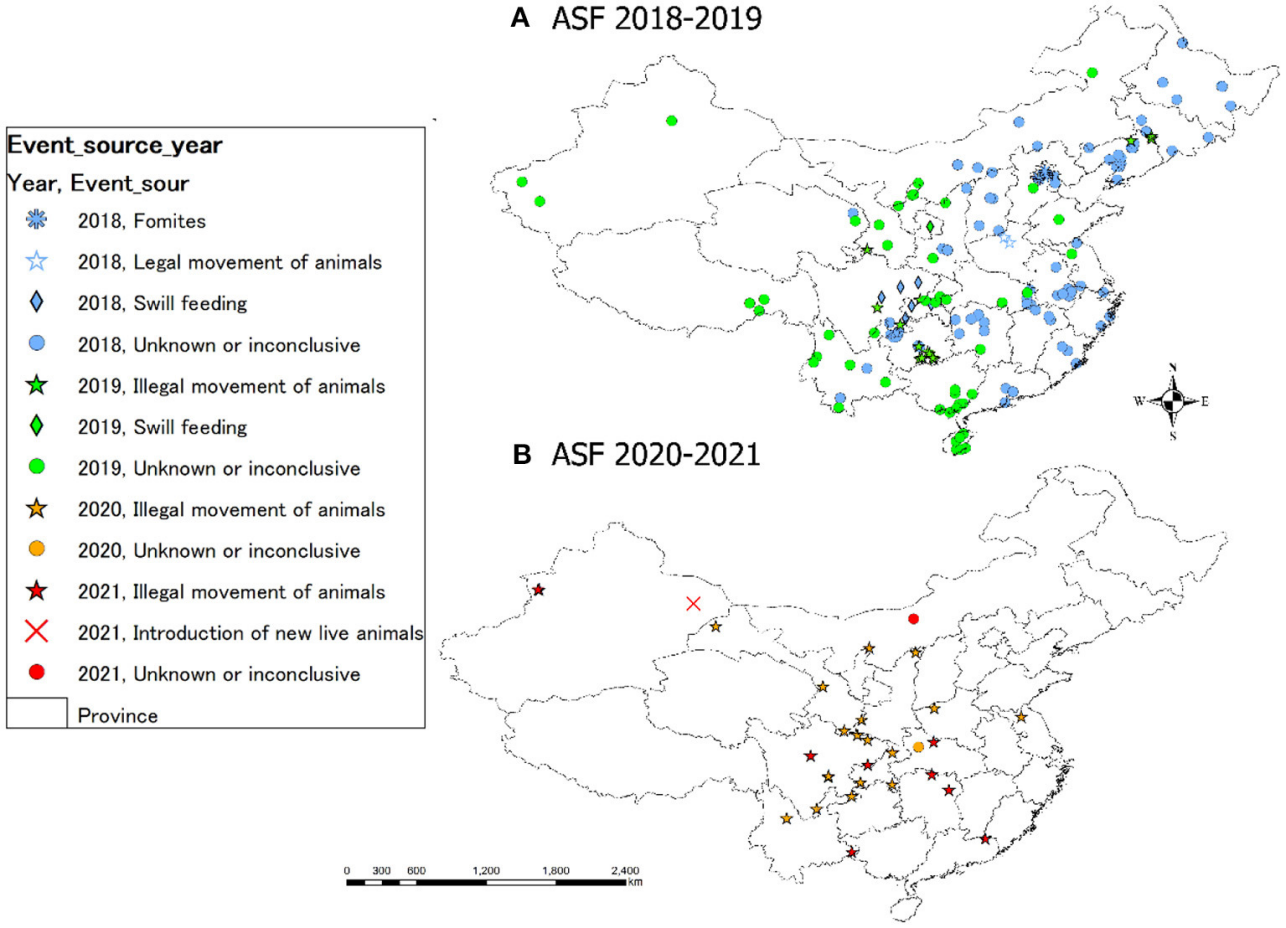
Spatial distribution of ASF in China. Each ASF notification was classified by event source; maps **(A)** and **(B)** show the situation of outbreaks in 2018–2019 and in 2020–2021, respectively. These information sources are based on official reports to the OIE.

The spatial distribution of outbreaks was also remarkably different in 2018–2019 and in 2020–2021. From 2018 to 2019, outbreaks were widely distributed throughout the entire nation. Meanwhile, from 2020 to 2021, the outbreaks seem to be concentrated around the central part of the country (Figure 2).

### Risk of Lower Virulent ASFV Spreading Nationwide

The results of the multi-distance spatial cluster analysis indicated that 625.5 km was the maximum distance of significant spatial association between ASF notifications of domestic pigs in China during the study period.

**Figure 3:**
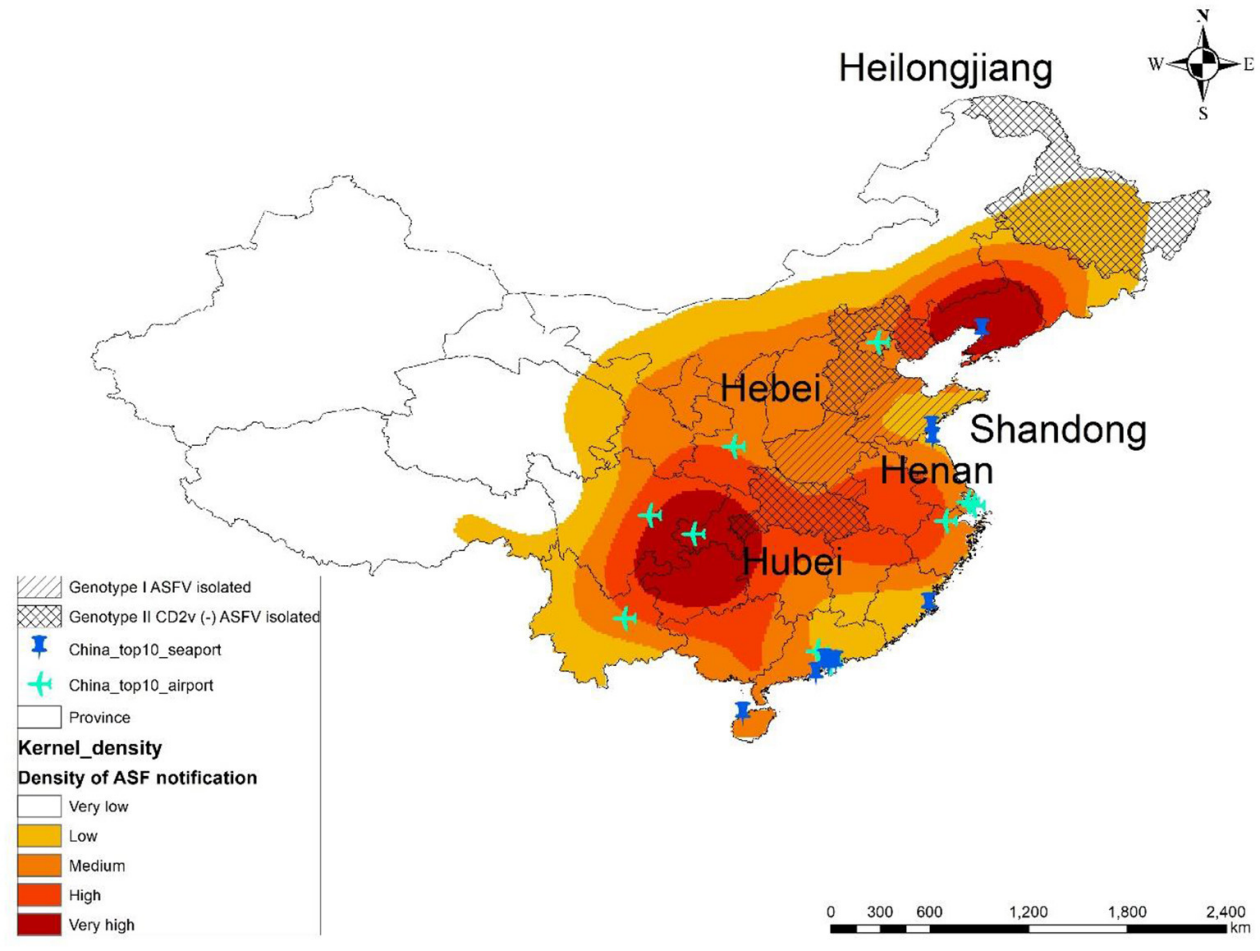
Density of ASF notifications in China overlapped with the lower virulent ASFV reported area and China's 10 largest airports and seaports. The graduated color shades illustrate the estimated kernel density of ASF notifications (notifications/km^2^). Each colored area indicates frequency of ASF notification number per >4.61 × 10^−5^ (very high), 3.11 × 10^−5^ to 4.6 × 10^−5^ (high), 1.81 × 10^−5^ to 3.1 × 10^−5^ (medium), 6.9 × 10^−6^ to 1.8 × 10^−5^ (low), and <6.9 × 10^−6^ (very low). Provinces where ASFV genotype I and CD2v (–) ASFV genotype II were isolated are represented by a diagonal line and a grid line, respectively. The locations of China's 10 largest airports and seaports are represented by airplane and pin symbols, respectively.

The results of the kernel density estimation analysis showed that the two highest kernel density areas were located in Northeast and Midwest China. In Midwest China, the third highest kernel density area was detected in the central coastal areas. Areas of medium kernel density were widely distributed around the two main hot spot areas (Figure 3).

Among the provinces of high kernel density, five of them are also high swine production area (Sichuan, Yunnan, Guangxi, Hunan, and Hubei) (Supplementary Figure 1).

Four of the five provinces where the lower virulent ASFV was isolated were located in areas of medium or higher kernel density. In particular, Hubei province, where the lower virulent ASFV genotype II was reported in 2020, was located between the two main hotspots. On the other hand, the kernel density in Heilongjiang province, where seven lower virulent ASFV genotype II isolates were reported, was classified as low (Figure 3).

### The Risk of ASFV Spread From China

Of China's 10 largest airports, one is located in the “very high” risk area, followed by two in the “high”-risk, five in the “medium”-risk, and two airports in the “low”-risk area. In terms of China's 10 biggest seaports, one is located in the “very high”–risk area, followed by two in the “medium”, and seven seaports in the “low” kernel density area (Figure 3).

**Figure 4:**
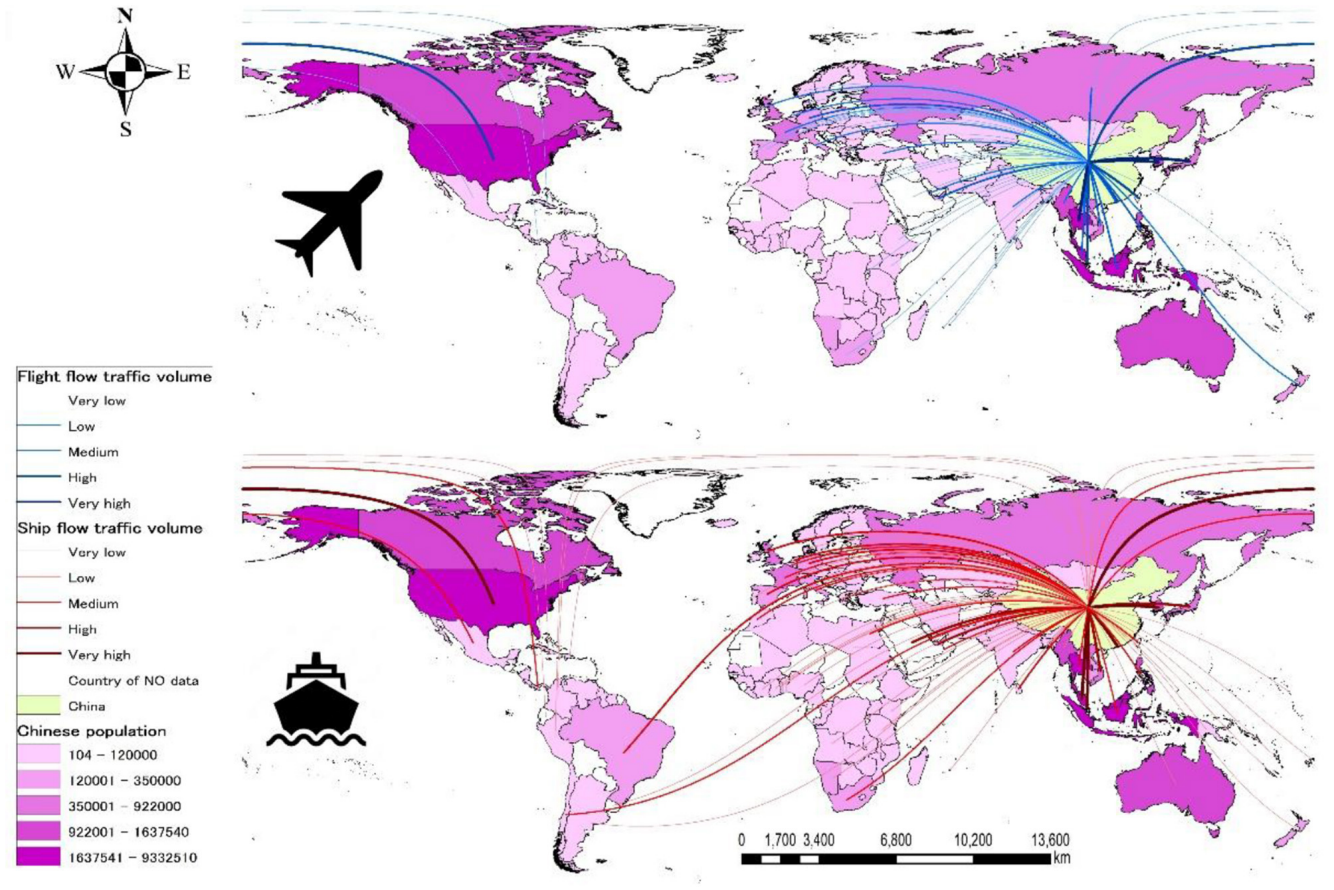
The flight and ship flows connected to China combined with the distribution of Chinese overseas residents. The flight flow and ship flow from China are depicted above and below, respectively. The graduated color and width in the map represent the total traffic volume from the highest (darker/thicker) to the lowest (lighter/narrower). Each line indicates total traffic volume of >1.81 × 10^7^ (very high), 8.91 × 10^6^ to 1.8 × 10^7^ (high), 2.91 × 10^6^ to 8.9 × 10^6^ (medium), 9.51 × 10^5^ to 2.9 × 10^6^ (low), and <9.5 × 10^5^ (very low) seats in flight flow. For ship flow, each line indicates total traffic volume of >7.51 × 10^6^ (very high), 3.61 × 10^6^ to 7.5 × 10^6^ (high), 2.21 × 10^6^ to 3.6 × 1 0^6^ (medium), 7.51 × 10^5^ to 2.2 × 10^6^ (low), and <7.5 × 10^5^ (very low) per quarterly deployed capacity (TEU). In each map, the graduated color represents the number of Chinese overseas residents from the highest (darker) to the lowest (lighter).

There are 67 countries and regions that had flight routes from China in 2019. Of these countries, 31 belonged to Asia, 22 to Europe, eight to Africa, four to North America, and two to Oceania (Figure 4). In terms of the amount of flight flow, 81.1% of total flight capacity is in Asia, 11.1% in Europe, 6.3% in North America, 0.9% in Africa, and 0.6% in Oceania (Supplementary Table 1).

In Q1 of 2020, China had ship flow to 81 countries and regions. Of those countries, 26 belonged to Asia, 19 to Europe, 15 to Africa, eight to Oceania, seven to South America, and six to North America (Figure 4). In terms of the amount of ship flow, 63.9% of total capacity is in Asia, 15.8% in Europe, 9.9% in North America, 5.2% in Africa, 4.4% in South America, and 1.0% in Oceania (Supplementary Table 2).

The number of Chinese overseas residents per country is shown in Figure 3, showing that 71.4% of Chinese overseas residents (29,000,000) live in other countries of Asia, followed by 16.3% (6,604,000) in North America, 5.5% (2,230,000) in Europe, 3.7% (1,500,000) in Oceania, 1.7% (700,000) in Africa, and 1.4% (572,000) in South America.

## Discussion

Because the first ASF outbreak was reported in 2018 in China, continuous outbreaks have been reported in the country (9). In March 2020, 10 regulations were issued by the Chinese government to further strengthen the prevention and control of ASF and to strictly enforce illegal activities in the swine industry chain (43). The regulations are designed to restrict behaviors that pose a risk of spreading ASF (e.g., concealment of the outbreak, use of illegal vaccines, illegal transport of pigs, and swill feeding). The change in the spatiotemporal trend of outbreaks observed in 2018–2019 and in 2020–2021 (see Figures 1, 2) may be associated with the enforcement of this crackdown. However, there is no reliable and sufficient information on the contribution of the enforcement of this regulation to disease control and can therefore only speculate. Recent outbreaks have been confirmed mainly from the south-central area, and most of these were attributed to the illegal transport of animals (9). Considering that about 45% of this “illegal transport of animals” were reported at highway checkpoints and that the maximum distance associated between cases was calculated to be 625.5 km in this study, it is highly likely that pigs were transported from distant locations (9). Of the ASF outbreaks reported from China, 44 outbreaks (28, 15, and 1 cases reported in 2018, 2019, and 2020, respectively) have occurred in backyard farms, and seven cases were detected at slaughterhouses in seven different provinces (9). Before ASF emergence in China, more than 60% of pigs were produced by small-scale or backyard farmers, which generally have low biosecurity (44). Around 50% of these farmers still exist after the introduction of ASF (45, 46); hence, the potential for ASF to cause serious consequences remains high (44). Furthermore, the fact that ASF cases were reported from slaughterhouses indicates the potential risk of underreported outbreak, as well as the risk of contaminated pork being distributed. These multiple uncertainties suggest that unreported ongoing outbreaks might be present across the country.

In March 2020, the Harbin veterinary research institute released information about their ongoing ASF vaccine development (16). Around the same time, illegal vaccines, which appeared to be copied from the Harbin Institute vaccine, emerged in China (18, 47). In May 2021, the Harbin Institute reported that lower virulent ASFV of genotype II was isolated from samples collected at several locations in 2020 (13). Subsequently, the identification of ASFV strains belonging to genotype I, which were shown to cause chronic infections in affected animals, was reported from Shandong and Henan province in June 2021 (12). The potential circulation and spread of lower virulent viruses that cause chronic infections can be assumed to delay detection and thus complicate efforts to control ASF (12, 13). Under conditions where outbreaks are not effectively monitored, this could lead to further outbreaks, which could spread silently throughout the country, and also poses the risk of spreading the virus outside the country.

The results of the multi-distance spatial cluster analysis indicated that the maximum associated distance between ASF cases in China during this study period was 625.5 km. Long-distance transportation of live pigs is common in China, and the movement of infected pigs by truck is considered to be the main reason why ASF outbreaks spread quickly throughout the country in the early period (48). The reason that the maximum associated distance was extremely long can be attributed to this traditional transportation system.

The five provinces where lower virulent ASFV genotype I or genotype II was reported locate close to the hotspots of ASF notification. On the basis of the assumption of underreporting and illegal transportation across the country, it cannot be ruled out that such viruses are already spreading quietly to other parts of the country. In particular, Hubei province is in a high-risk area; therefore, it is more likely that lower virulent ASFV is prevalent in the surrounding areas. In Heilongjiang province, where the largest number of lower virulent ASFV isolates has been found, no official outbreaks have been confirmed since 2019, despite the fact that highly virulent ASFV genotype II isolates have also been reported by Sun et al. (13). Considering the geographical proximity to the hotspots of ASF notification and the fact that many outbreaks have been reported along the border with China in the adjacent Russian Far East region, it is highly likely that ASF exists in this area (49). There are still many questions concerning the factors that influence the occurrence of ASF in China. As suggested by the results of this study, it is possible that the scale of the swine industry is one of the risk factors for ASF outbreaks (Supplementary Figure 1). However, more extensive and comprehensive research is needed to further understand the risk factors. For example, it would be interesting to survey the pork supply routes to urban areas and the associated inter-provincial transportation networks. Differences in the surveillance effort between regions may be one of the social factors for reporting bias.

If the lower virulent virus in China is released from the country, then the control of ASF in the world would be extremely difficult. In this study, by analyzing the general risk of ASFV spreading from China, we also implied the risk of these lower virulent viruses spreading. Currently, our study showed that eight of the 10 largest airports in China locate in areas of medium to high risk. Three of the 10 largest seaports in China locate in the medium or higher risk areas. The relationship between the location of the port and the density of ASF notification of the surrounding area is not yet clear. However, it is reasonable to assume that people would use an airport close to their residence, and if the density of ASF notification in that area was high, then the risk of exposure to contaminated materials would be high (50).

Over 80% of flights from China were concentrated in Asia. In proportion to this, at airports in Asian countries, there have been many reports of ASFV detection in pork products illegally brought in from China by passengers (51–54), JAQS, (55). In view of this, the United States, where many flights arrive from China, and Central American countries, which have yet to experience an ASF outbreak, need to monitor the risk of ASF entry.

The same can be said for ship flow. Because information on the number of passengers on board was not available for this study, the flow of cargo ships was considered here. It is assumed that the more the ship is loaded, the more crew members will be on board. Stockpiling food for the long voyage is essential, and if the port is located in a high risk area, then the risk of the food being contaminated with the virus could increase. In addition, if leftover food is discarded at the destination, then the risk of ASFV introduction increases. In fact, it is believed that the ASF outbreak in Georgia in 2007 was caused by leftover food brought in from East Africa (56); therefore, this pathway of virus entry should not be ignored. However, it should be noted that there is a high degree of uncertainty in this regard. We have no information on how the pork value chain in China is organized. Investigating the domestic distribution channels for live pig and pork products (e.g., where the pork available on the market is produced and how food procurement provided to shipping companies and airlines is organized) would be an important point to more accurately assess the risk of ASF spread from the country. Compared to flights, the destinations of ships were more varied, with more routes to Africa and South America. Currently, an ASF outbreak was confirmed in the Caribbean country of the Dominican Republic in the summer of 2021 (9). Although the origin of the outbreak is still unknown, this fact once again emphasized the warning that diseases can jump to distant parts of the world. Meanwhile, the risk of the virus being reverse imported must also be considered. If there are traffic flows going to a destination, it means that there are flows coming back. These risks should also be carefully monitored, especially because there are various types of viruses circulating in Africa.

Although more than 70% of Chinese overseas residents are concentrated in Asia, it should be noted that even South America, which has the smallest number of Chinese overseas residents, has more than 500,000 people there. Those who reside there may have opportunities to bring back contaminated products from their home countries. In fact, there are already many Chinese pig farms in Africa, and plans are underway to build large-scale pig farms in South America as well (57, 58). In recent years, China has been actively investing in humanitarian aid and development projects overseas, and it is believed that many people have been going into the field from their home countries as a response (59). Because ASF has spread rapidly to other Asian countries after the disease entered China, it is essential to consider, in advance, various scenarios to prevent further outbreaks.

In this study, we highlighted the possibility of the lower virulent virus spreading throughout China and the general risk of ASFV release to the world based on the publicly available data source. However, it must be mentioned that our study was developed under several major biases and assumptions. For example, the outbreak-related data in this study are based on the information reported to the OIE-WAHIS database. This may not adequately reflect the actual situation although, because endemic countries are not obliged to report each outbreak to the OIE. Furthermore, the source of the outbreak was provided by the reported country, and the reliability of the information is not discussed. Another limitation is that the official reports did not contain any information about the lower virulent virus, so we had to refer to the only available research reports by Sun et al. (12, 13). Because of the partial coverage of the study area in these studies, potential biases existed, and thus, our approach was very limited. If a comparable study had been conducted at a nationwide level, then it might have provided a more accurate picture of the epidemic and, thus, might have provided different results. In response to the widespread of illegal vaccines, the Chinese government has tightened its crackdown on such vaccines (47). Various measures have been taken to control the outbreak, including the implementation of movement restrictions that divide China into five areas, along with unannounced inspections of slaughterhouses (60). However, as a country with a huge land area, it is quite difficult to control all activities. Accurate understanding of the situation is essential to control the outbreak. Outbreaks continue to occur because the circulation of the virus is being maintained somewhere. Although there are few cases reported from wild boar in Asia, wild boar are considered to be widely distributed and populations are also assumed to be high (61). Therefore, the trend of ASF cases in wild boar also needs to be monitored.

The most important aspect of controlling the disease is the same for both domestic pigs and wild boar: early detection. Especially in the case of the lower virulent virus reported in China, unclear clinical symptoms and a long incubation period make early detection of infected individuals more difficult (12, 13). It is important that China, as well as other countries, understands this and establishes comprehensive measures to control the disease. As an approach toward farmers, advising them on biosecurity should be a top priority. Furthermore, establishing an adequate compensation system for affected farmers would help prevent illegal trade (62). Farmers with little or no compensation could choose to hastily slaughter or sell their sick pigs at local markets (62), so regular ASFV testing at farms and slaughterhouses, as well as inspection of meat products on the market, would also help to monitor the situation.

Further research is needed to better understand the spread of ASF in China and other parts of Asia. The assessment developed here can be used as the basis for a detailed country assessment whenever accurate and complete data are available. Ultimately, we believe that it will contribute to improving the effectiveness of surveillance and control programs and epidemiological studies on this complex infectious disease.

## Data Availability Statement

The original contributions presented in the study are included in the article/Supplementary Material, further inquiries can be directed to the corresponding author.

## Author Contributions

SI, JB, and JS-V: conceptualization. SI and JB: methodology and formal analysis. SI, JB, MM-A, and JS-V: validation and writing— review and editing. SI, JB, and MM-A: writing—original draft preparation. JS-V: supervision. All authors contributed to the article and approved the submitted version.

## Funding

This research was funded by European Project H2020 VACDIVA—A Safe DIVA vaccine for African Swine Fever control and eradication, grant agreement no. 862874.

## Conflict of Interest

The authors declare that the research was conducted in the absence of any commercial or financial relationships that could be construed as a potential conflict of interest.

## Publisher's Note

All claims expressed in this article are solely those of the authors and do not necessarily represent those of their affiliated organizations, or those of the publisher, the editors and the reviewers. Any product that may be evaluated in this article, or claim that may be made by its manufacturer, is not guaranteed or endorsed by the publisher.
