# Supplementary material for: The Evolution of African Swine Fever in China: A Global Threat?
Source: Front Vet Sci. 2022 Mar 29;9:828498. doi: 10.3389/fvets.2022.828498 (PMC9001964; doi:10.3389/fvets.2022.828498)
Supplement: Supplementary file 1 [file Data_Sheet_1.docx]

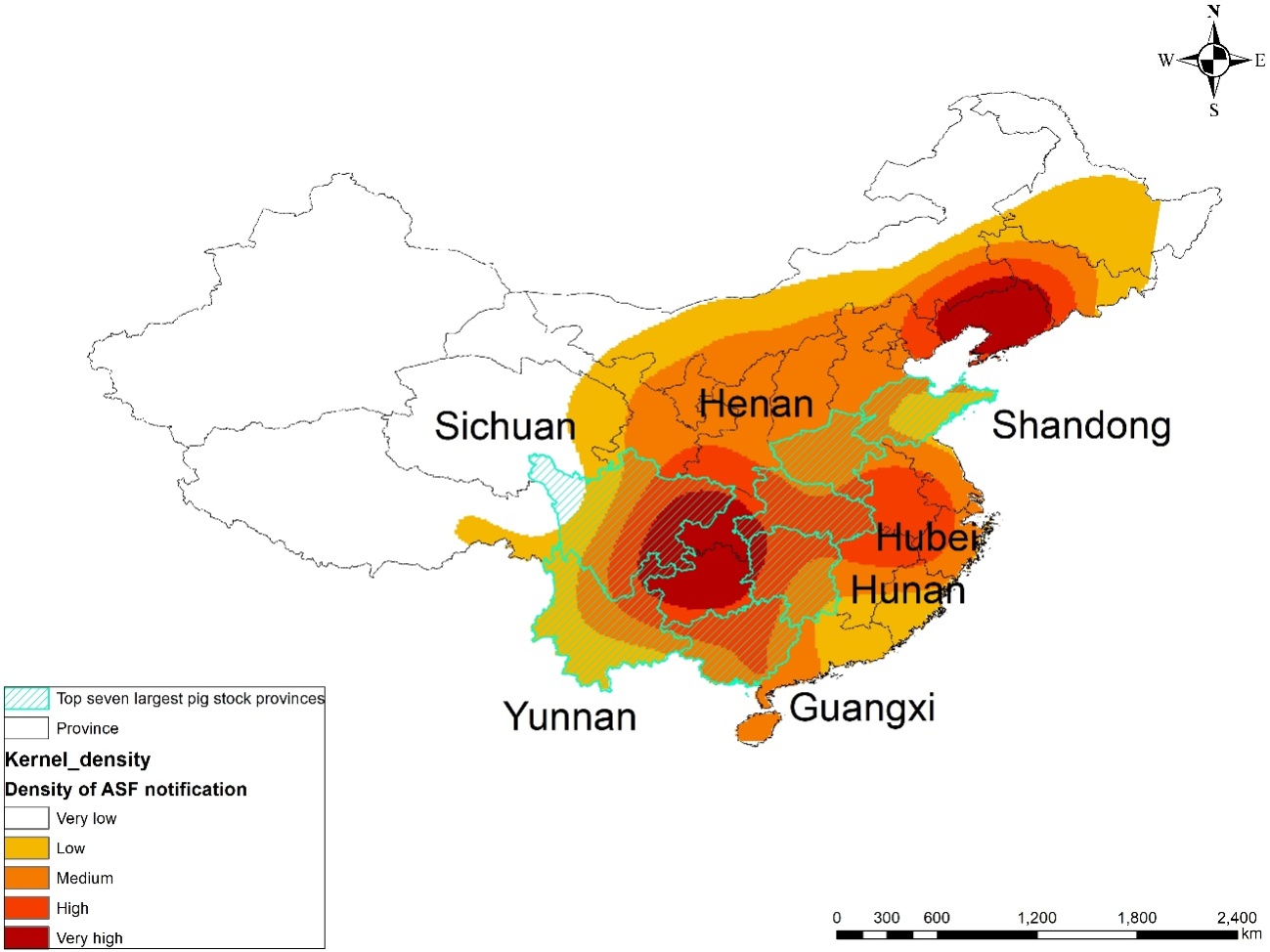


**Supplementary Figure 1. Relationship between Kernel density of ASF notification and pig industry in China.**The top seven swine production provinces (Henan, Sichuan, Hunan, Yunnan, Shandong, Hubei, and Guangxi) were overlaid with the Kernel density of ASF notification map.

Supplementary Table 1. Number of destination countries for flight flow by continent and their percentage of total capacity.

| Continent | No. of connected countries | Proportion of flows |
| --- | --- | --- |
| Asia | 31 | 81.1% |
| Europe | 22 | 11.1% |
| North America | 4 | 6.3% |
| Africa | 8 | 0.9% |
| Oceania | 2 | 0.6% |

Supplementary Table 2. Number of destination countries for ship flow by continent and their percentage of total capacity.

| Continent | No. of connected countries | Proportion of flows |
| --- | --- | --- |
| Asia | 26 | 63.9% |
| Europe | 19 | 15.8% |
| North America | 6 | 9.9% |
| Africa | 15 | 5.2% |
| South America | 7 | 4.4% |
| Oceania | 8 | 1.0% |
